# Identifying key genes in milk fat metabolism by weighted gene co-expression network analysis

**DOI:** 10.1038/s41598-022-10435-1

**Published:** 2022-04-27

**Authors:** Tong Mu, Honghong Hu, Yanfen Ma, Huiyu Wen, Chaoyun Yang, Xiaofang Feng, Wan Wen, Juan Zhang, Yaling Gu

**Affiliations:** 1 School of Agriculture, Ningxia University, Yinchuan, 750021 China; 2 Key Laboratory of Ruminant Molecular and Cellular Breeding, Ningxia Hui Autonomous Region, Ningxia University, Yinchuan, 750021 China; 3 Maosheng Pasture of He Lanshan in Ningxia State Farm, Yinchuan, 750001 China; 4 Animal Husbandry Extension Station, Yinchuan, 750001 China

**Keywords:** Genetics, Animal breeding

## Abstract

Milk fat is the most important and energy-rich substance in milk, and its content and composition are important reference elements in the evaluation of milk quality. However, the current identification of valuable candidate genes affecting milk fat is limited. IlluminaPE150 was used to sequence bovine mammary epithelial cells (BMECs) with high and low milk fat rates (MFP), the weighted gene co-expression network (WGCNA) was used to analyze mRNA expression profile data in this study. As a result, a total of 10,310 genes were used to construct WGCNA, and the genes were classified into 18 modules. Among them, violet (r = 0.74), yellow (r = 0.75) and darkolivegreen (r =  − 0.79) modules were significantly associated with MFP, and 39, 181, 75 hub genes were identified, respectively. Combining enrichment analysis and differential genes (DEs), we screened five key candidate DEs related to lipid metabolism, namely PI4K2A, SLC16A1, ATP8A2, VEGFD and ID1, respectively. Relative to the small intestine, liver, kidney, heart, ovary and uterus, the gene expression of PI4K2A is the highest in mammary gland, and is significantly enriched in GO terms and pathways related to milk fat metabolism, such as monocarboxylic acid transport, phospholipid transport, phosphatidylinositol signaling system, inositol phosphate metabolism and MAPK signaling pathway. This study uses WGCNA to form an overall view of MFP, providing a theoretical basis for identifying potential pathways and hub genes that may be involved in milk fat synthesis.

## Introduction

Milk is not only the source of nutrition for newborn cows, it is also an important source of protein, sugar, lipids, and other nutrients for humans^1^. Milk fat is the most important and energy-rich substance in milk and is an important component in the production of butter and yogurt, with a content of about 3–5% in milk. Milk fat also plays an important role in the nutrition and metabolism of human growth and development^2^ Polyunsaturated fatty acids such as conjugated and non-conjugated linoleic acid (C18:2) contained in milk fat play a beneficial role in lowering blood lipids, suppressing immune responses, and stimulating lipid metabolism^3^; while high concentrations of saturated fatty acids such as myristic acid (C14:0), lauric acid (C12:0) and palmitic acid (C16:0) increase the concentration of low density lipoproteins in the blood, which is associated with cardiovascular disease^4^. Therefore, the content and composition of milk fat is the main reference element to evaluate the quality of milk. Nowadays, milk fat content is not only one of the important indicators for the core competitiveness of dairy products, but also a major target trait for dairy cattle breeding^5^. Exploring the theory and methods of milk fat formation and regulation to improve milk fat content in dairy cows has become a hot spot in international lactation biology research.

In the past, many scholars have extensively studied the complex regulatory mechanisms of mammary gland development and elucidated the major pathways of milk fat synthesis (including de novo synthesis and FA uptake in the blood)^6^. Breeding researchers have identified a range of potent genes and biomarkers in milk fat metabolism with the widespread use of next-generation sequencing technologies and the dramatic reduction in sequencing costs^7^. Despite the transcriptome determining the characteristic of spatial and temporal specificity, there is less linkage to phenotypic data^8^. Weighted gene co-expression network analysis (WGCNA) can combine gene expression with phenotypic data^9,10^ and gather genes with similar expression patterns into one module^11^. Genes in modules are often involved in the same function or pathway, which can be used in data analysis of complex processes, and playing an important role in exploring the characteristics of gene networks associated with complex diseases^12^. For example, several biomarker genes screened and identified using the WGCNA method are associated with many biological problems such as cancer^13^, types I diabetes^14^, rheumatoid arthritis^15^, feed efficiency^16^, and meat quality^17^. Potential of this approach for grouping genes into the functional modules and revealing regulatory mechanisms underlying the complex traits have been highlighted in many recent studies^18^. In addition, there have been some studies on functional characteristics and lactation properties in ruminant mammary gland^19^, however, the application of WGCNA in milk fat metabolism in dairy cows has not been reported.

In this study, we used WGCNA to comprehensively analyze the mRNA expression profile data of high and low milk fat percentage (MFP) dairy cow mammary epithelial cells which measured by Illumina PE150 in the early stage of our group. Enrichment analysis of hub genes in modules closely related to MFP reveal its potential functions. In order to explore the signature genes and important functional enrichment pathways associated with MFP and provide a theoretical basis for understanding the complex biology of the milk fat synthesis process in dairy cows.

## Results

### Overview of BMECs sequencing data

Using Illumina PE150 sequencing platform, 81,605,996–97,102,888 and 78,710,246–88,676,080 raw_reads were obtained in high and low MFP BMECs, and 80,633,532–94,731,948 and 76,807,276–86,508,476 clean_reads were obtained after removing the adapter related, containing N and low quality, respectively. The sequencing error rate of the 8 samples is 0.02%, Q20 is greater than 97%, Q30 is greater than 94%, the GC content is about 53%, which ensures the accuracy of the subsequent analysis. The mapping rates for each sample after comparing clean_reads to the reference genome were 94.43% (H_2046), 94.96% (H_2098), 94.79% (H_2190), 94.97% (H_2226), 94.08% (L_2034), 94.32% (L_2037), 94.71% (L_2137) and 94.69% (L_2170).

### Principal component and correlation analysis of samples

**Figure 1:**
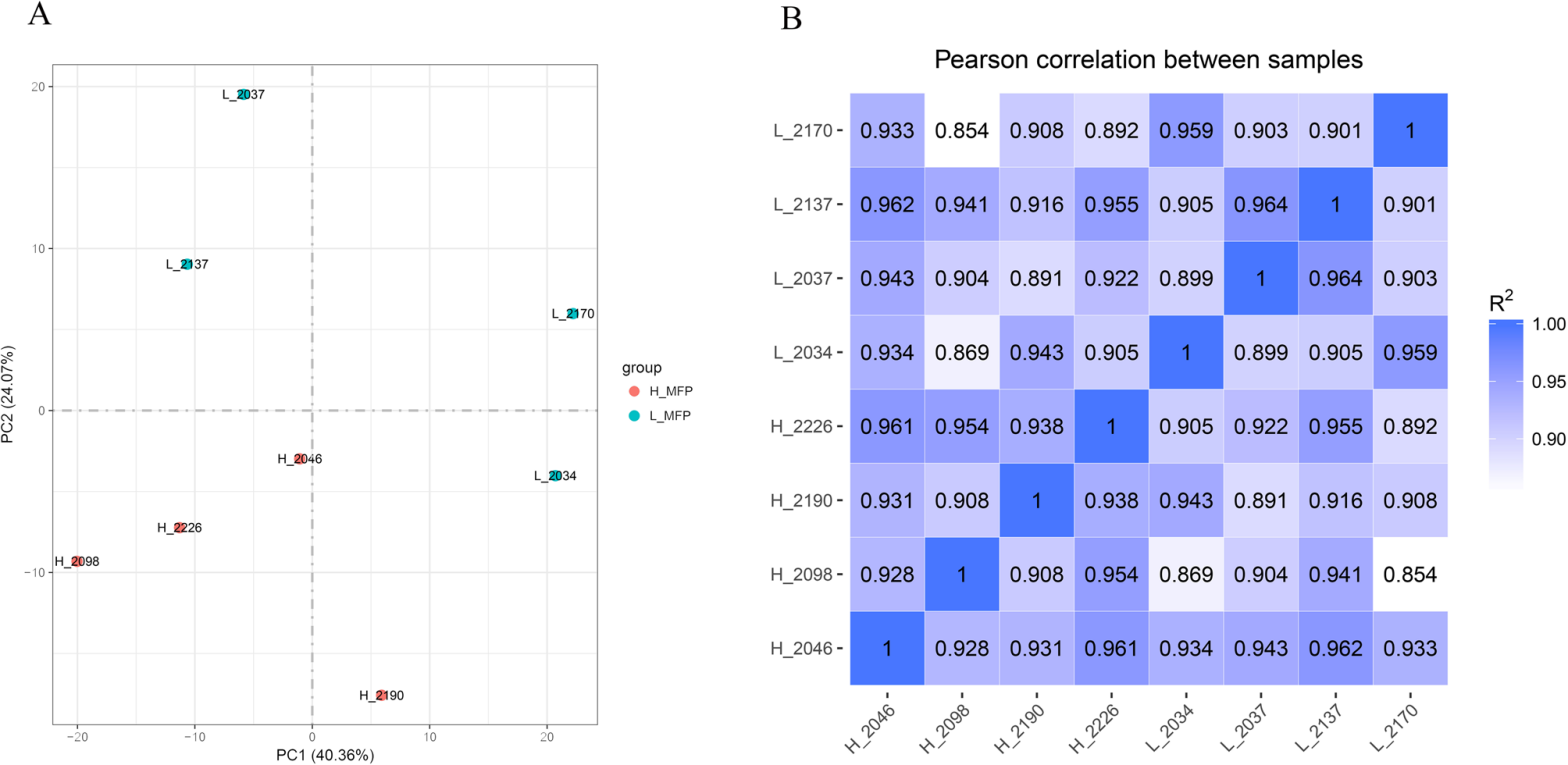
Principal components and correlation analysis. (**A**) Results of sample principal component analysis; (**B**) heat map of inter-sample correlation analysis.

The correlation of gene expression levels between samples and principal component analysis are important indicators to test the reliability of experimental samples. Principal component and correlation analysis was performed based on TPM values of all genes in each sample (Fig. 1). It was found that the samples of the high-milk fat group and the low-milk fat group were significantly different, and the correlation coefficients within the group were all above 0.89, which indicated a high similarity of expression patterns within the samples, and no outlier samples were found. Therefore, the transcriptome sequencing results are reliable and can be used for subsequent analysis.

### Weighted correlation network analysis

**Figure 2:**
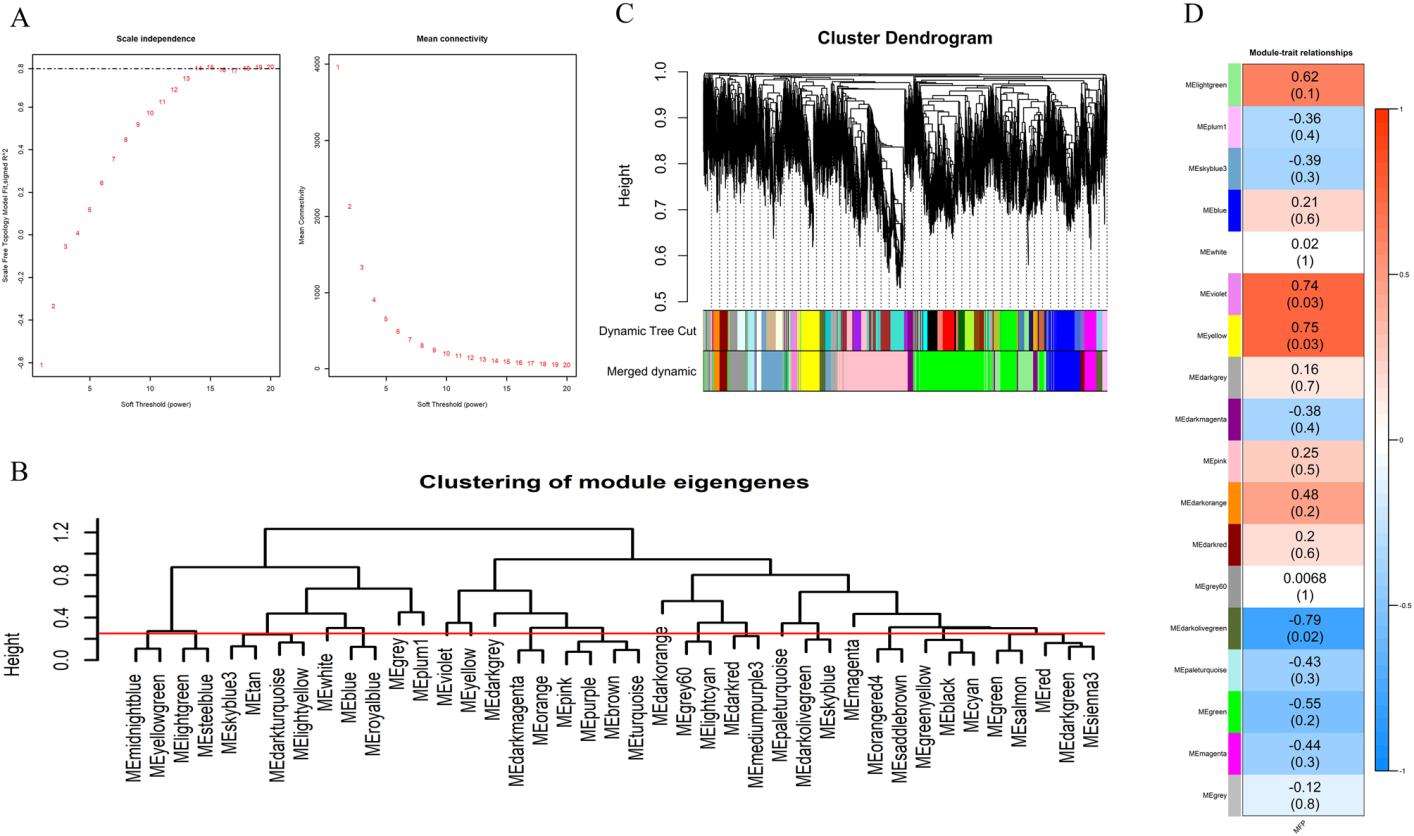
Weighted gene co-expression network analysis. (**A**) Analysis of the scale-free fit index for various soft-thresholding powers (left) and analysis of the mean connectivity for various soft-thresholding powers (right); (**B**) module clustering diagram, the height of the red line is 0.25, the modules below the line are the ones that are more similar and need to be merged; (**C**) gene dynamic shearing clustering tree, each color represents a module. The color in the first row is the result of the first clustering, and the color in the second row is the result of the modules after merging; (**D**) correlation analysis between module and MFP. Red represents positive correlation, green represents negative correlation, and the darker the color, the stronger the correlation.

WGCNA analyzed 10 310 genes obtained after data preprocessing. When the scale-free topology model fit reached 0.8 (R^2^ = 0.8), a soft thresholding power was 14 (β = 14) (Fig. 2A). The 41 co-expression modules were constructed by WGCNA (Fig. 2B), and 18 modules were obtained after merging modules with a similarity greater than 0.75 (Fig. 2C). The module containing the most genes was the green module (2 626 genes), followed by the pink module (1 890 genes), blue module (874 genes) and skyblue3 module (828 genes) (Supplementary Table 1).

**Figure 3:**
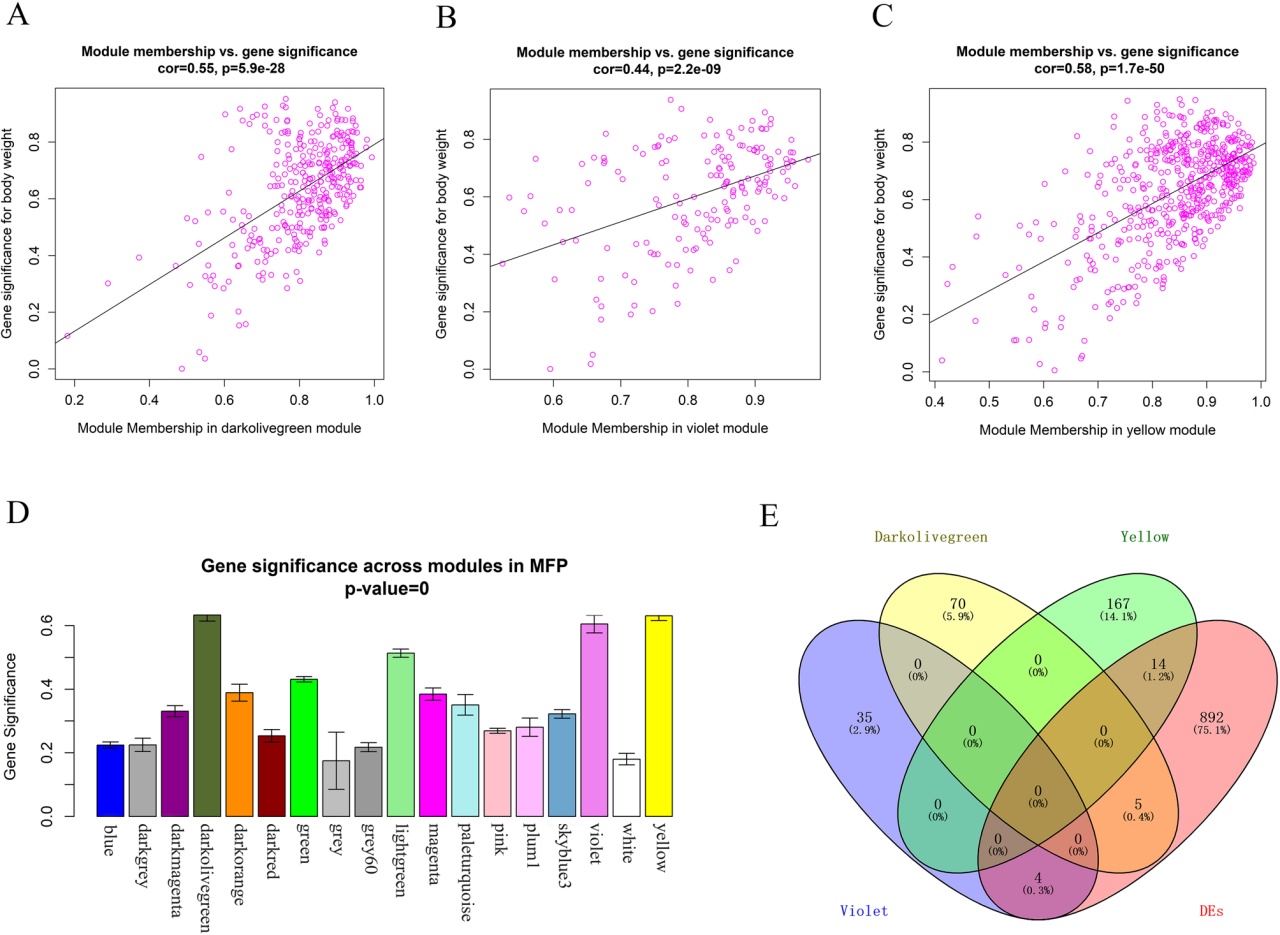
Major findings in module-trait correlation analysis. (**A–C**) A scatterplot of GS for MFP vs. MM in the darkolivegreen, violet and yellow module, respectively (a dot represents a gene); (**D**) module significance values of those co-expression modules associated with MFP (Module significance value indicated the summary of gene significance of all genes in each module, and different colors of column indicated different modules). (**E**) The number of DEs contained in the hub genes of the three modules.

The correlation between the co-expression module and the MFP phenotype was analyzed. The results showed that multiple modules were associated with MFP phenotype, among them violet (r = 0.74) and yellow (r = 0.75) modules are significantly positively correlated with MFP (Fig. 2D), including 169 genes (Fig. 3B) and 547 genes (Fig. 3C) respectively, while darkolivegreen module was significantly negatively correlated with MFP (r =  − 0.79) (Fig. 2D), including 336 genes (Fig. 3A).There were 39, 181 and 75 genes that had high gene significance (GS > 0.4, Fig. 3D) with MFP phenotype in these three modules, respectively, and the correlation between all these genes and module members (MM) is greater than 0.9, so these genes were considered as hub genes (Supplementary Table 2).

After obtaining hub genes of the three modules, the intersection was taken with the 915 DEs screened from the transcriptome data (Supplementary Table 3) (Fig. 3E), and it was found that the hub genes were isolated between the three modules, and the yellow module contains 14 DEs, which is the largest number, followed by the darkolivegreen module (5 DEs) and the violet module (4 DEs). The DEs contained in each module are shown in Table 1.

**Table 1:** DEs contained within the darkolivegreen, violet and yellow modules.

Modules	Symbol	Gene_locus	Log_2_FoldChange	*P*-value
Yellow	SLC34A2	6:45,185,925–45,205,946	8.646257	0.002225
	IL23R	3:78,077,155–78,150,856	8.425257	0.039538
	SYTL5	X:105,235,090–105,341,728	8.268285	0.040235
	DMKN	18:46,167,026–46,178,470	7.077906	0.000224
	TLR7	X:130,769,575–130,786,365	6.663896	0.003289
	POLQ	1:65,944,699–66,053,204	1.552549	0.01384
	SBSN	18:46,186,110–46,190,724	2.949797	0.012799
	PSTPIP2	24:45,737,786–45,832,060	1.563937	0.013285
	CIP2A	1:53,314,658–53,338,782	1.029365	0.046888
	ATP8A2	12:33,380,874–33,831,448	− 13.011	4.14E-07
	TMEM215	8:11,330,165–11,335,552	− 2.16016	0.031349
	SPON1	15:38,457,984–38,773,001	− 1.34677	0.003535
	PLCXD2	1:56,425,478–56,476,144	− 0.99338	0.043642
	PI4K2A	26:18,819,244–18,845,948	− 0.94566	0.011456
Darkolivegreen	UNC5D	27:31,082,652–31,370,431	10.0263	0.010558
	LCN2	11:98,781,893–98,785,927	8.328422	0.000447
	ERH	10:81,172,307–81,184,975	0.925145	0.027718
	SLC16A1	3:30,382,285–30,411,742	0.78718	0.017597
	FAM171B	2:9,553,295–9,633,357	− 3.41115	0.001731
Violet	ZNF365	28:18,471,094–18,494,821	8.432485	0.012442
	VEGFD	X:128,109,577–128,153,168	8.164288	0.003258
	ID1	13:61,179,938–61,182,003	4.735816	0.000109
	MOXD1	9:70,257,747–70,355,603	2.521412	0.002972

### Functional enrichment analysis of hub genes

**Figure 4:**
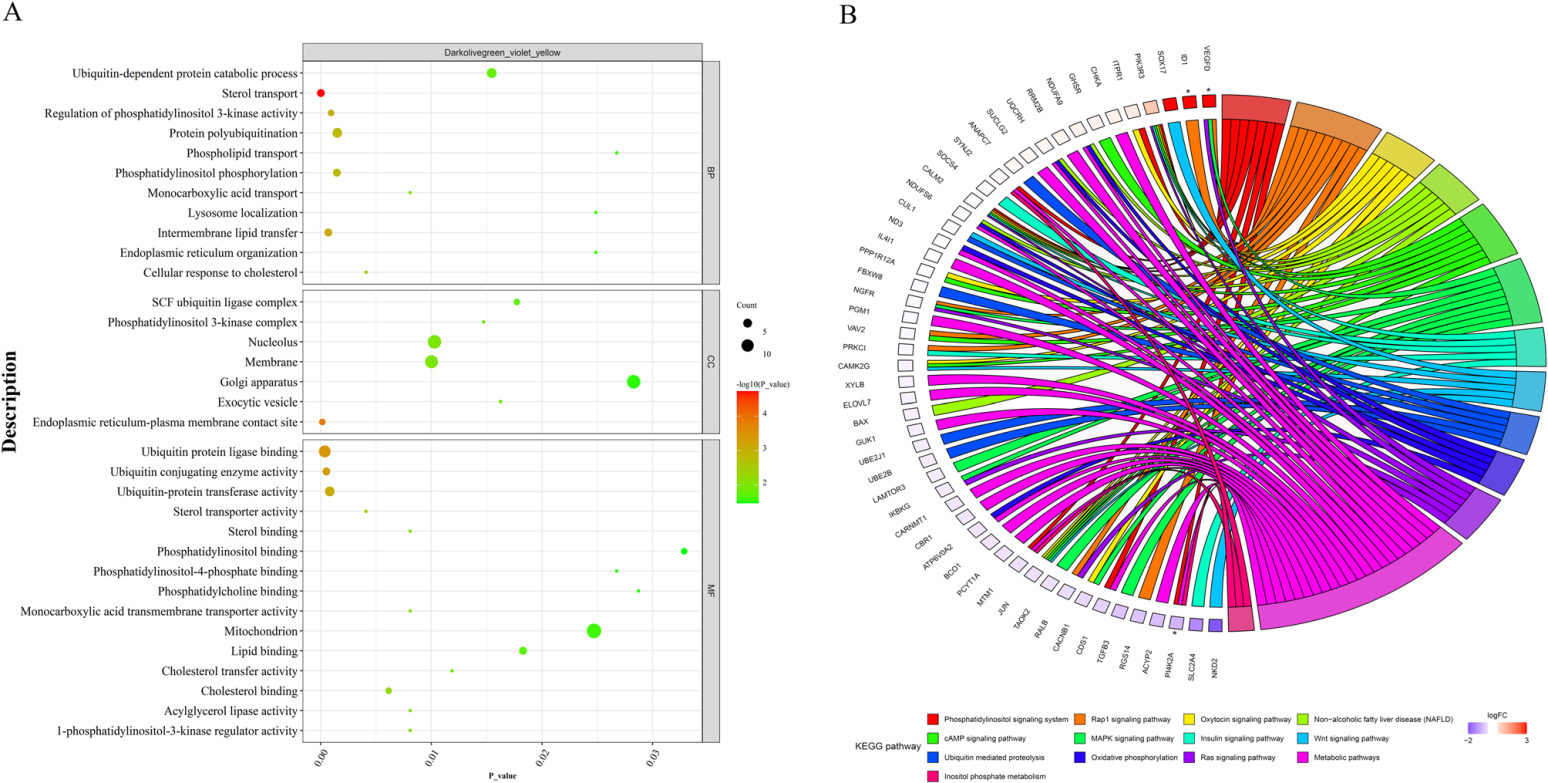
Enrichment analysis of hub genes. (**A**) Bubble plot of GO enrichment analysis of hub genes; (**B**) Circos plot of KEGG enrichment analysis of hub genes, with *for DEs.

To determine the specific functions of the genes within the three modules significantly associated with MFP, we performed GO and KEGG enrichment analysis on 295 hub genes in the three modules. A total of 1 301 GO terms were enriched as a result (Supplementary Table 4), and 180 GO terms were significantly enriched (*P* < 0.05). There were 37 significantly enriched GO terms related to lipid metabolism, and Fig. 4A shows 11 significantly enriched biological processes (BP) related to lipid metabolism, 15 molecular functions (MF) related to lipid metabolism and 7 representative cellular components (CC). GO terms closely related to milk fat synthesis include acylglycerol lipase activity, ubiquitin protein ligase binding, intermembrane lipid transfer and lipid binding. Notably, SLC16A1, ATP8A2 and PI4K2A are hub genes related to lipid metabolism and also DEs screened in the transcriptome data (*P* < 0.05), which were mainly enriched in monocarboxylic acid transport, phospholipid transport and phosphatidylinositol phosphorylation terms.

**Figure 5:**
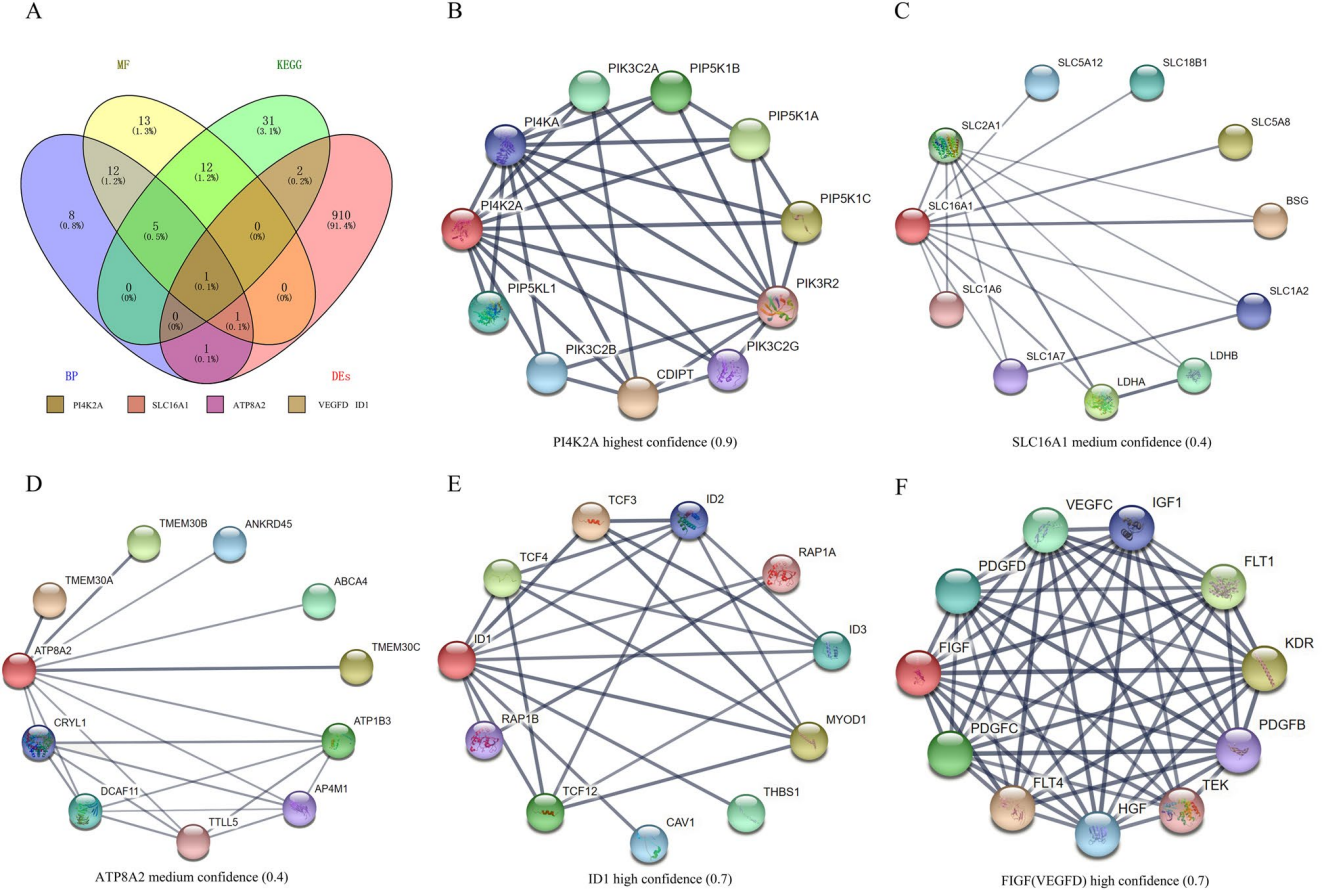
Protein interaction network analysis of key candidate genes for milk fat metabolism. (**A**) DEs contained in hub genes related to lipid metabolism significantly enriched in BP, MF and KEGG pathways; (**B–F**) protein interactions network plots for PI4K2A, SLC16A1, ATP8A2, ID1 and VEGFD, respectively; Thicker lines indicate stronger data support.

**Figure 6:**
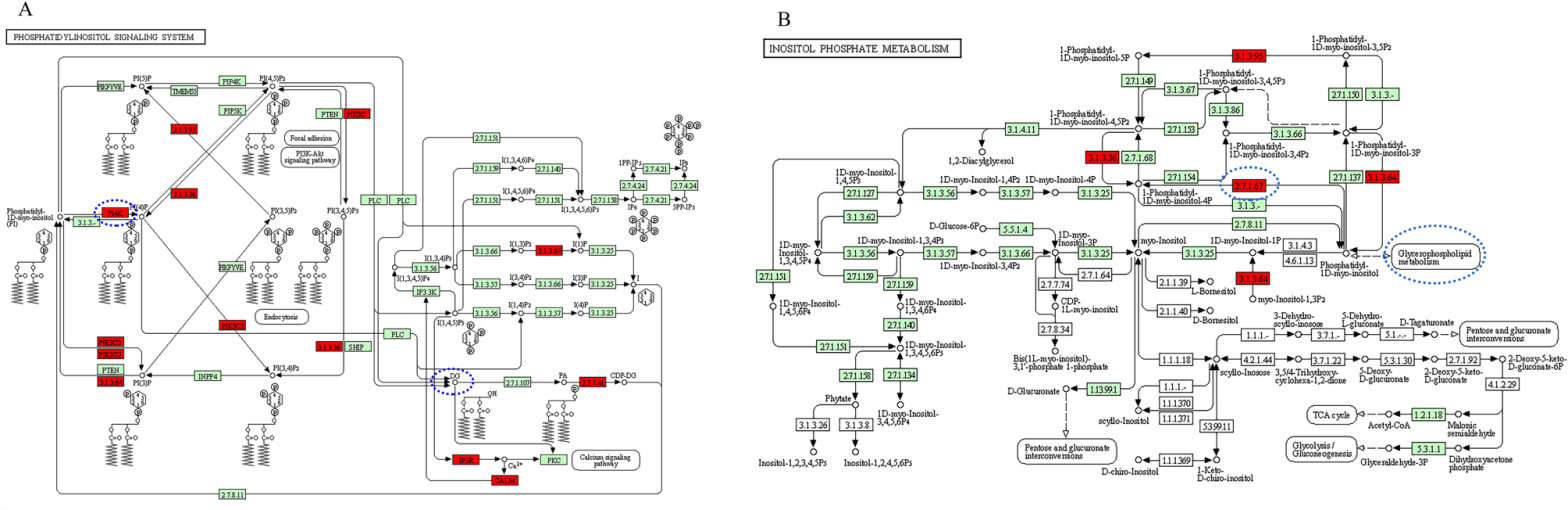
Localization of the DEs PI4K2A in the phosphatidylinositol and inositol phosphate metabolic pathways. (**A**) Phosphatidylinositol signaling system; (**B**) inositol phosphate metabolism; Blue dashed boxes indicate the specific location of the PI4K2A gene and the pathway involved in triglyceride synthesis.

The KEGG enrichment results showed that 239 pathways were enriched (Supplementary Table 4), among them 66 pathways were significantly enriched (*P* < 0.05) and 13 pathways were associated with lipid metabolism. Figure 4B show the hub genes contained in 13 lipid metabolism-related pathways, with PI4K2A, VEGFD and ID1 being the DEs and significantly enriched to phosphatidylinositol signaling system, inositol phosphate metabolism, Rap1, MAPK and Ras signaling pathway. Interestingly, the down-regulated gene PI4K2A within the yellow module was significantly enriched in GO terms and KEGG pathways related to lipid metabolism (Fig. 5A). The PI4K2A gene is involved in diacylglycerol and glycerophospholipid metabolism of the phosphatidylinositol and inositol phosphate metabolic pathways, respectively (Fig. 6), which suggesting that PI4K2A may play an important function in milk fat synthesis. In addition, some hub genes were not significantly different between the high and low milk fat groups, but they were significantly enriched in the GO terms and KEGG pathways related to lipid metabolism (Table 2), and these genes are likely to play a potential role in milk fat synthesis.

**Table 2:** Hub genes significantly enriched to lipid metabolism-related GO terms and the KEGG pathway (non-DEs).

Genes	Enrichment type	Gene_locus	Log_2_FoldChange	*P*-value
SOCS4	BP, MF, KEGG	10:67,504,612–67,518,536	0.045374504	0.915092
UBE2J1	BP, MF, KEGG	9:60,811,836–60,836,538	− 0.220599808	0.598978
PIK3R3	BP, MF, KEGG	3:99,963,094–100,063,831	0.899633498	0.138561
UBE2B	BP, MF, KEGG	7:45,944,974–45,957,591	− 0.227826355	0.484955
CUL1	BP, MF, KEGG	4:111,931,406–112,019,124	0.030709711	0.947533
GRAMD1B	BP, MF	15:34,027,052–34,225,316	− 0.488085938	0.475434
GRAMD1A	BP, MF	18:45,743,703–45,767,909	− 0.697366096	0.347438
HDAC6	BP, MF	X:86,916,119–86,933,774	0.057863262	0.911513
SLC16A7	BP, MF	5:53,698,770–53,892,992	0.838823738	0.493724
OSBPL5	BP, MF	29:48,450,137–48,518,334	− 0.267683419	0.465346
OSBPL9	BP, MF	3:94,541,126–94,702,551	− 0.017030062	0.9537
PLEKHA8	BP, MF	4:66,266,959–66,326,201	− 0.263741351	0.438704
NDFIP2	BP, MF	12:54,578,637–54,649,183	0.06042112	0.852244
KLHL42	BP, MF	5:82,047,183–82,063,595	0.467789955	0.244411
AKTIP	BP, MF	18:21,848,713–21,860,197	− 0.113342608	0.744733
UBE2T	BP, MF	16:80,305,103–80,309,731	− 0.263593638	0.649904
USP15	BP, MF	5:51,147,112–51,275,743	0.082023217	0.816405
NKD2	MF, KEGG	20:71,307,541–71,311,025	− 2.162984035	0.21443
PCYT1A	MF, KEGG	1:70,824,603–70,877,084	− 0.382732935	0.245659
MTM1	MF, KEGG	X:33,290,880–33,359,619	− 0.388458912	0.381421
FBXW8	MF, KEGG	17:58,026,797–58,141,074	− 0.082095548	0.835147
ITPR1	MF, KEGG	22:21,523,823–21,876,681	0.305321904	0.491774
JUN	MF, KEGG	3:87,265,962–87,268,007	− 0.389295398	0.532967
BAX	MF, KEGG	18:55,531,246–55,534,932	− 0.200972783	0.794355
IKBKG	MF, KEGG	X:37,587,308–37,604,668	− 0.264005172	0.577697
RALB	MF, KEGG	2:71,941,425–72,015,169	− 0.439258818	0.228456
NGFR	MF, KEGG	19:37,093,181–37,112,123	− 0.096890779	0.978203
SUCLG2	MF, KEGG	22:33,859,633–34,134,874	0.124067981	0.680863
RRM2B	MF, KEGG	14:62,133,632–62,171,095	0.143114079	0.752695

### Protein interaction network analysis

The results of hub genes enrichment analysis intersected with DEs to identify SLC16A1, ATP8A2, PI4K2A, VEGFD and ID1 may be the key candidate genes involved in milk fat synthesis (Fig. 5A). We performed protein interaction network analysis on these five candidate genes. Since these genes are not directly functionally related to each other, we have selected the top 10 interacting proteins that are similar in function to the candidate gene. PI4K2A and VEGFD (FIGF) had the highest strength of data support with the 10 proteins with a common function (Fig. 5B,F) and the confidence levels of 0.9 and 0.7 respectively, followed by ID1 with a confidence level of 0.7 (Fig. 5E). The strength of data support for SLC16A1 and ATP8A2 with the top 10 proteins was low (Fig. 5C,D), with moderate confidence (0.4), but still have reliable reference value.

### Tissue expression profile analysis of key candidate genes

**Figure 7:**
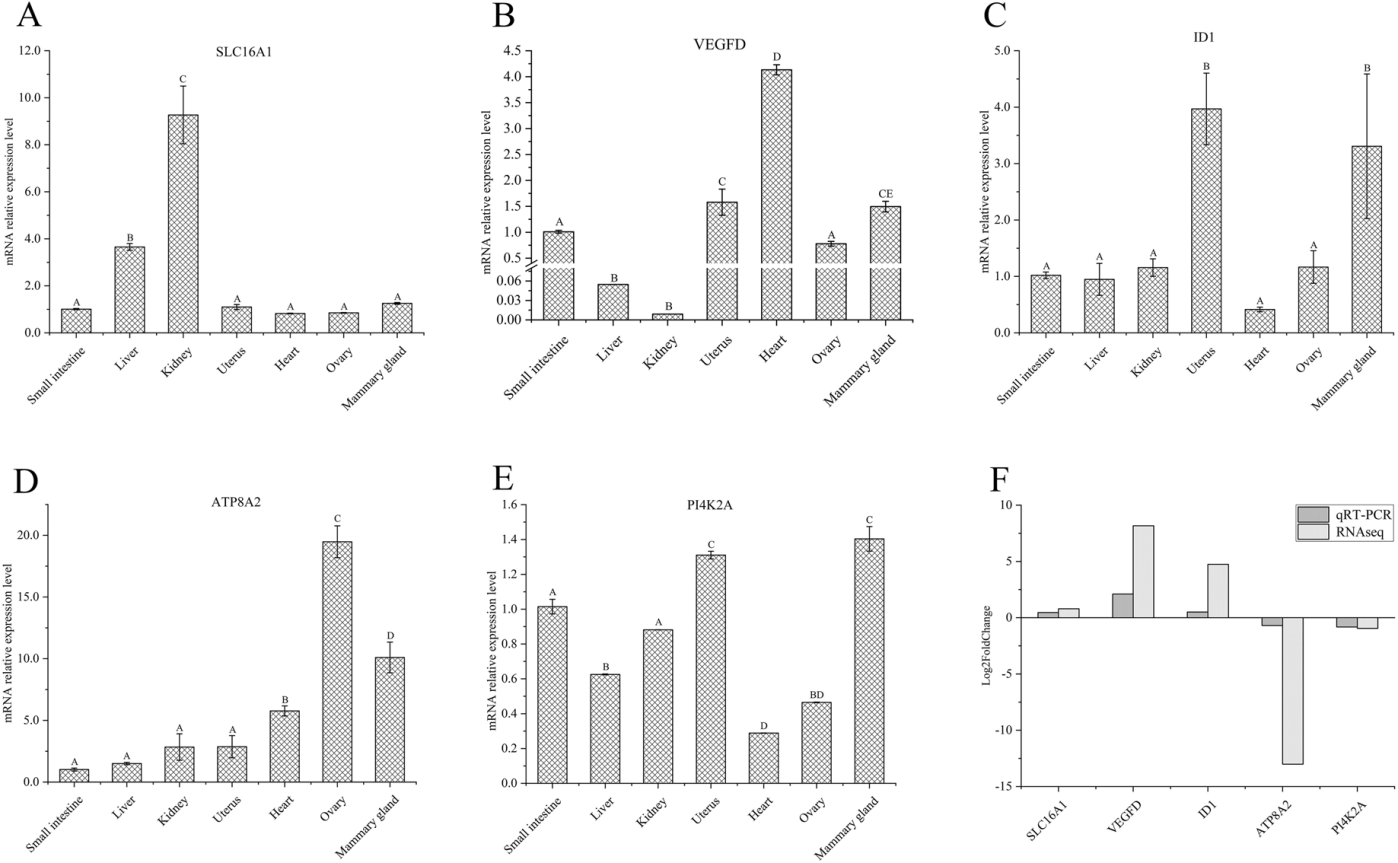
Expression levels of key candidate DEs in milk fat metabolism. (**A–E**) Expression levels of SLC16A1, VEGFD, ID1, ATP8A2 and PI4K2A genes in different tissues, respectively; (**F**) expression of SLC16A1, VEGFD, ID1, ATP8A2 and PI4K2A genes in BMECs of high and low milk fat groups. Different capital letters indicate significant differences.

The expression levels of SLC16A1, ATP8A2, PI4K2A, VEGFD and ID1 varied in different tissues. PI4K2A gene expression was relatively highest in the mammary gland (Fig. 7E), which significantly higher than small intestine, liver, kidney, heart and ovary tissues (*P* < 0.05), and slightly lower in uterus than mammary gland. The expression level of ATP8A2 and ID1 genes in mammary gland ranks second (Fig. 7C,D); VEGFD gene expression in mammary gland was significantly lower than heart and similar to that in uterus (Fig. 7B). The SLC16A1 gene was highly expressed in the kidney, followed by the liver, with lower expression in other tissues and non-significant differences (Fig. 7A). In addition, we examined the relative expression levels of key candidate genes for milk fat synthesis in BMECs from the high and low milk fat groups (three technical replicates), it was found that the trends of the qRT-PCR experiment results and the RNAseq sequencing results are consistent by using log_2_FoldChange to convert the difference multiples (Fig. 7F), confirming the accuracy of the transcriptome sequencing.

## Discussion

Some new analytical methods are gradually making up for the limitations of traditional biological research with the continuous innovation of sequencing technology and the rapid development of bioinformatics, which can fully and effectively explore the biological significance of the massive amount of data^20,21^. Compared to other regulatory networks, WGCNA is an effective data mining method that modularizes large datasets based on similar expression patterns of genes to obtain co-expression modules with high biological significance^22^. In recent years, WGCNA has been applied to explore the characteristics of human and plant life activities^23–25^. However, the use of WGCNA on the metabolism of milk fat has not been reported. Milk fat synthesis in dairy cows is related to many physiological and metabolic changes. To gain new insights into the expression and regulation of key genes in milk fat synthesis, we used WGCNA to comprehensively analyze the mRNA expression profile data of high and low MFP dairy cow mammary epithelial cells which measured by Illumina PE150 in the early stage of our group. Clustering the important genes into modules of specific biological pathways that may be associated with MFP in cows, thereby improving the efficiency of identification of important genes.

In this study, we constructed the first gene co-expression network for high and low MFP in Holstein cows, and obtained three modules significantly associated with MFP, violet, yellow and darkolivegreen respectively. The hub gene enrichment analysis in the three modules showed that SLC16A1, ATP8A2, VEGFD, ID1 and PI4K2A genes, which overlap with DEs, were significantly enriched to lipid metabolism-related pathways. Among them, the SLC16A1, ATP8A2 and PI4K2A genes were significantly enriched for monocarboxylate transport, phospholipid transport and phosphatidylinositol phosphorylation terms. It is well known that many monocarboxylic acids were utilised by the body’s metabolism, and acetic acid and β-hydroxybutyric acid are the main substrates for the de novo synthesis of fatty acids in ruminants and are essential for meeting the energy requirements (70%) and milk fat synthesis in cows. Acetic acid and β-hydroxybutyric acid play a positive regulatory role in the de novo synthesis of fatty acids, the transport and desaturation of long-chain fatty acids and the synthesis of triglycerides^26–28^; Phosphatidylinositol is a phospholipid, it is also one of the five main polar lipids in milk (less than 2% of total fat) in milk^29^. Polar lipids are the main component of the milk fat globule membrane (MFGM), which is responsible for wrapping the lipid droplets secreted by BMECs^30^. Therefore, SLC16A1, ATP8A2 and PI4K2A may be key candidate genes for the regulation of milk fat synthesis. The MAPK signalling pathway plays a key role in the inflammatory response and induces the expression of a variety of inflammatory mediators and pro-inflammatory cytokines^31^. The RAP1 pathway is a key component of the BMECs^32^, and during peak lactation in dairy cows, MAPK and RAP1 signaling pathways can increase milk production by regulating the proliferation and differentiation of BMECs^33,34^. Rap1 has been shown to antagonize Ras signals in inactive complexes by capturing its effector protein (serine/threonine kinase Raf)^35^. In this study, KEGG enrichment analysis revealed that PI4K2A, VEGFD and ID1 genes (i.e. DEs and hub genes) were significantly enriched in the phosphatidylinositol, Rap1, MAPK and Ras signaling pathway, which suggested that PI4K2A, VEGFD and ID1 genes were likely to be involved in the lactation process of cows. Notably, the PI4K2A gene was significantly enriched to the GO terms and KEGG pathways associated with lipid metabolism and was involved in diacylglycerol and glycerophospholipid metabolism in the phosphatidylinositol and inositol phosphate metabolic pathways, respectively, which indicates its potential to be involved in regulating milk fat synthesis.

PI4K2A is a key enzyme for the synthesis of phosphatidylinositol 4-phosphate with multiple cell signaling functions^36^, which is critical for epidermal growth factor receptor degradation^37^, transferrin receptor recycling^38^, autophagy-lysosome fusion^39^ and prognosis of breast cancer patients^40^. A genome-wide association study of milk fatty acid composition in Italian Simmental and Italian Holstein cows by using single nucleotide polymorphism arrays^41^, which revealed that PI4K2A may be involved in milk fat metabolism. In addition, the CDIPT gene, which significantly interacts with PI4K2A, and it also plays an important role in fatty acid and energy metabolism^42^, which reflecting the potential importance of the PI4K2A gene in milk fat metabolism. This study found that the expression level of PI4K2A was significantly higher in dairy cows’ mammary tissue than the small intestine, liver, kidney, heart and ovary. It further indicates that PI4K2A may have an important function in milk fat synthesis in dairy cows, and a more in-depth functional verification of specific mechanisms is required. DNA binding inhibitor 1 (ID1) is a helix-loop-helix transcription factor that is highly expressed in brown adipose tissue^43^ and promotes obesity by inhibiting brown fat thermogenesis and white fat browning^44^. Functionally ID1 is involved in regulating the transcriptional activity of ADD1/SREBP-1c, thereby regulating adipogenesis^45^. Marcin et al.^46^ showed that Mammalian target of rapamycin can regulate mammary epithelial cells growth through ID1. ATP8A2 is a P4-ATPase that transfers (flips) phosphatidylserine and phosphatidylethanolamine from the ectoplasmic lobules of the cell membrane lipid bilayer to the cytoplasmic lobules, resulting in asymmetric lipid partitioning between membrane lobules^47^. Vascular endothelial growth factor D (VEGFD) is considered the main angiogenic component of adipose tissue^48^, which enhances lymphangiogenesis and reduces obesity-related immune accumulation in mouse adipose tissue^49^, but VEGFD deficiency does not affect adipose tissue development in mice^50^. The expression levels of ATP8A2, ID1 and VEGFD were significantly higher in mammary tissue than small intestine, liver, kidney and ovary of dairy cows, which suggested that they may have potential biological functions in the mammary gland. SLC16A1 has an important role in short-chain fatty acid transport^51^. Hu et al.^52^ studies suggested that SLC16A1 may be involved in hepatic lipid metabolism in pigs, which is consistent with the high-level expression results of SLC16A1 in liver tissues of this study. Although the expression level of the SLC16A1 gene in dairy cows’ mammary gland tissue is significantly lower than that in liver and kidney. However, compared with other tissues, the expression abundance of SLC16A1 is still at the upper-middle level, so the role of SLC16A1 in dairy cows’ milk fat metabolism cannot be ignored. In the future, members of our group will continue to investigate the functional mechanisms of these key candidate DEs (SLC16A1, VEGFD, ID1, ATP8A2 and PI4K2A) in lipid metabolism screened by WGCNA, and in order to elucidate their specific regulatory mechanisms.

## Conclusion

In this study, a comprehensive analysis of mRNA expression profile data based on Illumina PE150 sequencing of high and low MFP BMECs was performed by WGCNA, resulting in three modules (violet, yellow and darkolivegreen) that were significantly associated with MFP. After enrichment analysis, a total of 5 candidate DEs related to lipid metabolism were screened out, namely PI4K2A, SLC16A1, ATP8A2, VEGFD and ID1. Among them, PI4K2A is more likely to be involved in milk fat metabolism. The results of this study provide a new way to understand the function of genes in milk fat synthesis in dairy cows and it also provide a new perspective on the study of the lactation process in cattle.

## Materials and methods

### Ethics statement

Animal experiments were conducted in accordance with the Regulations for the Administration of Affairs Concerning Experimental Animals (Ministry of Science and Technology, China, 2004). It is authorized by the Animal Ethics Committee of Ningxia University (permit number NXUC20200619). The cattle used in the experiments was electric shocked before being released. Take tissue samples immediately, making all efforts to minimize its suffering. This work also conformed to the requirements of American Veterinary Medical Association (AVMA) Guidelines. This study is reported in accordance with the recommendations put forward by the ARRIVE guidelines.

### Data source and preprocessing

The data of 14 543 mRNA expression profiles in this study were obtained from the results of Illumina PE150 sequencing of BMECs of high and low MFP cows in the previous study by our research group (Supplementary Table 5). Sequencing samples were obtained from the Maosheng pasture of He Lanshan in Ningxia state farm, where the test cows were fed the same balanced total mixed diet. A total of 245 Holstein cows of similar age and in the mid and late lactation were selected. Collect milk samples of each cow in the morning, at noon, and in the evening for dairy herd improvement (DHI). Screen 8 Holstein cows with somatic cell counts within 100,000/mL and extreme differences in MFP (Table 3). BMECs were isolated from fresh milk by aseptic collection^53^, and the library construction and transcriptome sequencing were carried out by Beijing Nuohe Zhiyuan Biotechnology Co., Ltd.

**Table 3:** High and low MFP of Holstein cattle.

Item	Number	Age	Lactation days	MFP (%)	SCC (100,000/mL)
High-milk fat group	H_2098	29.81	186	4.82	5
	H_2046	30.57	189	4.54	2
	H_2226	28.56	160	4.74	9
	H_2190	28.89	157	4.88	5
Low-milk fat group	L_2034	30.57	187	2.60	6
	L_2037	30.5	175	2.81	5
	L_2170	30.43	189	2.85	8
	L_2137	29.41	150	2.84	7

*Number* the number of each cow, *MFP* milk fat percentage, *SCC* somatic cell count, *age* month age of cattle at the time of sampling.

A chain-specific library was constructed by removing ribosomal RNA. After passing the library inspection, Illumina PE150 sequencing was performed. After the original data is obtained, the reads with adapter, N (undetermined base information) ratio greater than 0.002, and low-quality bases with a read length of more than 50% are removed. After sequencing error rates (Q20 and Q30) and GC content distribution checks, clean reads for subsequent analysis were obtained. Hisat2 (http://ccb.jhu.edu/software/hisat2, version 2.1.0) software were used to compare and analyze the RNA sequencing (RNA-seq) data (the reference genome version is bos_taurus_Ensembl_97)^54^.

Since the mRNA expression profile data in transcriptome sequencing is represented by the FPKM value, the FPKM was converted to TPM by using the colSums function of the tidyverse package of the R (version 4.1.1). After that, the principal component and correlation analysis of the eight samples was performed by online post-sale tool platform provided in Beijing Nuohe Zhiyuan Biotechnology Co., Ltd (https://magic.novogene.com/customer/ main#/home/2d9dc26d1e059b931b9ac5364 9482c7c).

### Construction of co-expression network

The median absolute deviation of different gene expression profiles were first calculated by the apply function in R to eliminate outliers and abnormal values in the data set, and then the goodSamplesGenes function was used to detect missing values and samples below the sample threshold. And finally, 10,310 genes with relatively high expression were obtained. The co-expression network of mRNA expression profile data was constructed by the R package WGCNA^55^. The construction of a weighted gene network requires the optimal selection of soft thresholding power β that improves co-expression similarity and calculates the adjacency. Therefore, picking the optimal soft thresholding power β was performed using the function pickSoftThreshold (based on the criterion of approximate scale-free topology) in the R package WGCNA. When 0.8 is used as the correlation coefficient threshold (R^2^ = 0.8), the soft thresholding power β was 14 and the minimum number of genes in the module is 111, and the number of genes to construct the co-expression network is set to 100. The module detection sensitivity was 2 (deepSplit = 2), and the cut height for merging of modules was 0.25 (mergeCutHeight = 0.25, i.e., merge into one module if the correlation coefficient of eigengenes within the module is greater than 0.75).

### Identification of key candidate genes

In the module-trait correlation analysis, hub genes were considered as genes with GS greater than 0.4 and high module group members (MM, weighted correlation index > 0.9), indicating a significant correlation with milk fat percentage.

### Functional enrichment and protein interaction network analysis

Here, functional enrichment analysis was performed using the KOBAS website (http://kobas.cbi.pku.edu.cn/genelist/, version 3.0) and its results were visualized by the R package GOplot (version 1.0.2). The hub genes were intersected with the differential genes (DEs) screened by the transcriptome (*P* < 0.05) and combining the results of enrichment analysis to screen key candidate genes for milk fat metabolism, and protein interaction network analysis carried by String website (https://www.string-db.org/https://www.string-db.org/, version 11.5).

### qRT-PCR validation of key candidate genes

Small intestine, liver, kidney, heart, ovary, uterus and mammary gland tissues were collected from three cows in the mid and late lactation with similar age, and the tissues were cut and quickly placed in liquid nitrogen and brought back to the laboratory for total RNA extraction and first-strand cDNA synthesis. Real-time quantitative reverse transcription PCR (qRT-PCR) was used to detect the expression of key candidate genes for milk fat in different tissues and to verify their expression levels in BMECs of high and low MFP cows.

Total RNA was extracted by using RNA simple Total RNA Kit (Tiangen Biochemical Technology Co., Ltd). First-strand cDNA synthesis was performed by using PrimeScript RT Kit (Takara, Dalian, China). qRT-PCR (three replicates) was performed by SYBR Premix Ex Taq II (TaKaRa, Dalian, China) on the Bio-Rad CFX96 Touch Real-Time PCR Detection System (Bio-Rad, Hercules, CA, USA). Amplification procedure: 95 °C for 30 s, 95 °C for 5 s, annealing for 30 s, 40 cycles. qRT-PCR primers were designed by using Primer Premier 5.0 and the primers span at least one intron, the sequence and annealing temperature of each primer were shown in Table 4.

**Table 4:** Primer sequence and annealing temperature.

GenBank ID	Genes	Primer sequence (5′–3′)	Product length/bp	Tm/℃
NM_001100316.1	PI4K2A F	ATCCGCAACACTGATCGAGG	137	60
	PI4K2A R	AGCCCATTATCTATGGCCGC		
NM_001101043.2	VEGFD F	CCACTCGCAGGAATGGAAGATCAC	238	62
	VEGFD R	GAAAGGGGCATCTGTCCTCACA		
NM_001037319.1	SLC16A1 F	TGGCAGCACCTTTATCCTCTAC	162	60
	SLC16A1 R	ACTCCACAATGGTCACCAATCC		
NM_001097568.2	ID1 F	CTGGGATCTGGAGTTGGAGC	155	59
	ID1 R	GGAACACACGCCGCCTCT		
NM_001163802.3	ATP8A2 F	GCCCACAGCTGGAGAAGATA	189	60
	ATP8A2 R	GTACTTGGCCGTGCTGATCT		
NM_001034034.2	GAPDH F	TCGGAGTGAACGGATTCGG	192	60
	GAPDH R	TGATGACGAGCTTCCCGTTC		

### Statistical analysis

The statistical significance of differences between the two groups was analyzed using a non-parametric test or t-test based on the data distribution characteristics. All the analyses were conducted using the software R; the *P* < 0.05 was considered statistically significant. The relative expression of DEs was analyzed by the 2^−ΔΔCt^ method and normalized using the glyceraldehyde-3-phosphate dehydrogenase (GAPDH) gene.

### Institutional review board statement

The Animal Ethics Committees of Ningxia University approved the experimental design and animal sample collection for the present study (permit number NXUC2 0200619). And animal experiments were conducted strictly followed the guidelines of the Regulations for the Administration of Affairs Concerning Experimental Animals (Ministry of Science and Technology, China, 2004).

## Supplementary Information

Supplementary Legends.

Supplementary Table 1.

Supplementary Table 2.

Supplementary Table 3.

Supplementary Table 4.

Supplementary Table 5.

## Data Availability

All data generated or analyzed in this study are included in this article [and its Supplementary Information File], and the datasets have been submitted to the SRA database with the Accession Number PRJNA730595. Access to the data of permanent link to https://www.ncbi.nlm.nih.gov/sra/PRJNA730595.
