# Supplementary material for: Identifying key genes in milk fat metabolism by weighted gene co-expression network analysis
Source: Sci Rep. 2022 Apr 27;12:6836. doi: 10.1038/s41598-022-10435-1 (PMC9046402; doi:10.1038/s41598-022-10435-1)
Supplement: Supplementary file 1 — Supplementary Legends. [file 41598_2022_10435_MOESM1_ESM.docx]

**Supplementary table**

**Supplementary table 1.** Details of the 18 modules

**Supplementary table 2.** Details of the hub genes within the three modules significantly associated with MFP

**Supplementary table 3.** Details of differential genes

**Supplementary table 4.** Detailed results of the hub genes enrichment analysis

**Supplementary table 5.** The data of 14 543 mRNA expression profiles
